# Clinical Vignettes: Integrated Care of Cancer Patients by Oncologists and Cardiologists

**DOI:** 10.2174/157340311799960654

**Published:** 2011-11

**Authors:** Erin Hofstatter, Hamid Saadati, Kerry Russell, Raymond Russell

**Affiliations:** 1 Section of Medical Oncology, Yale University School of Medicine; 2 Smilow Cancer Hospital; 3 Section of Cardiovascular Medicine, Yale University School of Medicine

**Keywords:** Cardiotoxicity, anthracyclines, trastuzumab, left ventricular ejection fraction.

## Abstract

Because of the paucity of large, randomized trials concerning the cardiac care of patients with cancer, treatment and prevention of chemotherapy-induced cardiotoxicity must rely on insights gained from small trials and case reports as well as the application of guidelines developed for the general population. In these clinical vignettes, we present patients referred by their oncologists to a cardiologist for specialized evaluation and management of cardiotoxicity with the goal of emphasizing the importance of identifying risk factors for cardiotoxicity, initiating evidence-based therapy, and establishing a close collaboration between oncologists and cardiologists.

## INTRODUCTION

Patients undergoing chemotherapy treatment for cancer face a variety of potential therapy-related risks and side effects. In recent years, cardiovascular complications due to chemotherapy have been increasingly recognized. Such complications can range in severity from asymptomatic transient arrhythmias to irreversible myocardial necrosis, vasospasm-related myocardial ischemia, or pericarditis. Though the anthracycline class of compounds and trastuzumab are agents most frequently associated with cardiotoxicity, virtually any chemotherapeutic drug has the potential to cause cardiovascular side effects; patients with underlying heart disease are particularly vulnerable [1]. 

Given the complexity of balancing competing oncologic and cardiac risks, many cancer patients may benefit from a multidisciplinary approach to their care. Here we present three clinical vignettes which demonstrate how the integration of oncology and cardiology can enhance overall care for patients undergoing treatment for cancer.

## CLINICAL CASE 1

EW is a 50-year-old female with a history of asthma and hypothyroidism who initially presented two years previously with a two-month history of increasing chest and back pain. Chest x-ray revealed a right lateral chest wall/pleural-based soft tissue mass with underlying fifth rib destruction and compression fractures of T9 and T11. Computed tomography of the chest revealed a 3.9 x 3.5 cm irregular left breast mass, bilateral axillary and subcarinal lymphadenopathy and widespread bony metastases predominately in the thoracic spine and sternum. Biopsy revealed invasive ductal breast cancer, which was estrogen receptor (ER)-positive, progesterone receptor (PR)-negative, and HER-2/neu-positive. 

Prior to the initiation of chemotherapy for metastatic breast cancer, the patient’s echocardiogram revealed a left ventricular ejection fraction (LVEF) of 60%. She then received a standard loading dose of trastuzumab followed by combined treatment with trastuzumab and paclitaxel. After three months of therapy, repeat echocardiography revealed a decrease in her LVEF to 39-45%. Of note, the patient had smoked a half a pack of cigarettes a day for 30 years. She also had a history of cocaine and alcohol abuse. Her family history was unremarkable.

Cardiology consultation was sought to assist in management of the patient’s decline in LVEF, in light of the treating oncologist’s preference to continue HER-2/neu-targeted therapy for as long as possible during the course of treatment for metastatic breast cancer. .

## CARDIOLOGY CONSULT QUESTIONS

### 1. How Frequently Does Cardiac Imaging Need to be Done While a Patient is on Trastuzumab? Is there an Advantage to Using one Imaging Modality Over Another?

Because of the potential for cardiac toxicity, frequent assessment of LVEF is recommended. Though no formal guidelines or consensus statements currently exist regarding cardiac monitoring during trastuzumab, most experts recommend that left ventricular function be evaluated prior to the initiation of trastuzumab therapy to establish the baseline LVEF, and that LVEF should be reassessed every three months during trastuzumab therapy [2]. In addition, many experts recommend that LVEF be measured every six months for two yearsafter completion of treatment with trastuzumab [2]. There are currently no data to guide consideration of less frequent or eventual cessation of LVEF monitoring in an asymptomatic patient with metastatic disease receiving trastuzumab on an indefinite basis. 

In regard to LVEF screening modality, there is no evidence to suggest definitive superiority of either echocardiography or equilibrium radionuclide angiography (ERNA/ MUGA) for the purposes of monitoring cardiac function in the setting of chemotherapy. Both echocardiography and ERNA are acceptable modalities provided that a quantitative assessment of LVEF is performed so that small changes can be detected over time. Echocardiography may be more appropriate in patients who require additional assessment of valve function, diastolic function, cardiac chamber sizes, pericardial effusions, or regional wall motion. Echocardiography may also be more appropriate in those patients who will require frequent or ongoing monitoring in an effort to minimize exposure to radiation, given that the amount of total body irradiation delivered with each ERNA has been estimated by some to be as high as the equivalent of 100-200 chest x-rays [3]. Conversely, ERNA may be preferred in patients who have limited windows for echocardiography as a result of significant obesity, COPD, or petite stature with narrow intercostal spaces.Because of variability in the results of LVEF assessment between echocardiography and ERNA, it is best to utilize the same modality in the serial monitoring of left ventricular function.

### 2. What is the Clinical Management of a Decrease in LVEF in a Patient on Trastuzumab? How Should this Affect Consideration for Additional Trastuzumab?

The cardiotoxicity associated with trastuzumab is generally a type 2 cardiotoxicity; that is, a reversible decline in left ventricular function that is not caused by cardiomyocyte necrosis or the development of fibrosis [2]. As a result, one of the mainstays of management of a decrease in LVEF during trastuzumab therapy is to discontinue therapy and reassess left ventricular function four weeks later. In addition, initiation of standard heart failure therapy with ACE inhibitors, beta-blockers and diuretics should be considered.

Once left ventricular function normalizes, trastuzumab therapy can be reinitiated, in part because the cardiotoxic effects of the agent do not appear to be a function of the cumulative dose. However, closer monitoring (every 2-4 weeks) of LVEF is warranted [4]. In rare circumstances, the LVEF suppression caused by trastuzumab can be irreversible. If the LVEF does not normalize after four to eight weeks, permanent discontinuation of trastuzumab therapy should be considered. In addition, if the LVEF decreases to a level of moderate-to-severe left ventricular dysfunction (LVEF<35%) or the patient develops clinical manifestations of decompensated heart failure, trastuzumab should not be restarted [5].

### 3. Should Trastuzumab be Avoided in Someone with Risk Factors for Cardiac Disease (Tobacco use, Family History of Cardiac Disease, etc)?

Age greater than 50 years, along with preexisting left ventricular dysfunction and treatment with anthracylines, are risk factors for the development of trastuzumab-associated cardiotoxicity [6]. However, there are currently no data to suggest that classic risk factors for coronary artery disease, other than advanced age, place women at greater risk for cardiotoxicity from trastuzumab [7]. An interesting question is whether chemotherapy, including trastuzumab-based chemotherapy, increases the risk of coronary artery disease based on changes in risk factor profile [8], and studies are currently being performed to address this important issue. However, as part of routine health maintenance, modifiable cardiac risk factors should be controlled in patients undergoing cancer therapy.

## CLINICAL CASE 2

DD is a 61-year-old female with hypertension, hypercholesterolemia and borderline diabetes who was diagnosed with an invasive carcinoma after routine mammography revealed an abnormality in the left breast. She ultimately underwent left mastectomy with axillary dissection, revealing a 1.6 cm, poorly differentiated, infiltrating ductal carcinoma with four of eight lymph nodes positive for metastatic carcinoma. The lesion was ER/PR-positive and HER-2/neu-negative. Prior to initiation of chemotherapy, she had undergone exercise stress testing with perfusion imaging, which revealed normal perfusion and left ventricular function. She also had a baseline ERNA study with an LVEF of 51%.She subsequently started standard adjuvant chemotherapy treatment with doxorubicin (60 mg/m^2^) and cyclophosphamide, given once every two weeks. However, after completion of four planned cycles of chemotherapy, the patient reported progressive dyspnea on exertion and tachycardia to her oncologist. Cardiology consultation was requested to assess her symptomatic dyspnea, both in the context of her recent course of anthracycline chemotherapy, and given that she was otherwise due to start taxane-based adjuvant chemotherapy.

The patient recalled that she first noted dyspnea on exertion after the first cycle of chemotherapy. She was a lifelong non-smoker and drank only an occasional glass of wine. Her family history was unremarkable for cardiac disease. At the time of her cardiac evaluation, examination revealed a normal jugular venous pressure with mild hepatojugular reflux. The patient was mildly tachycardic and her lung exam revealed bibasilar crackles. An EKG showed sinus tachycardia and mild T-wave flattening in the inferolateral leads. Echocardiography revealed a mild to moderately depressed LVEF of 40% and mild global hypokinesis. 

## CARDIOLOGY CONSULT QUESTIONS:

### 1. How Common is it to See a Drop in the LVEF After a Course of Anthracyclines? How Common is Cardiac Dysfunction After Only One Dose of Doxorubicin?

Anthracycline-based chemotherapy is associated with a risk of cardiotoxicity that is related to the cumulative dose. For example, the cumulative percentage of patients demonstratinga significant decline in LVEF who received 100 mg/m^2^ of doxorubicin is 0.5% and increases to 8.8% in patients who received 250 mg/m^2^ of doxorubicin [9]. This represents a type 1 cardiotoxicity that is a result of myocyte death and is therefore not reversible [2]. Quite rarely, transient left ventricular dysfunction can occur following a single dose of doxorubicin [10, 11], suggesting that type 2 cardiotoxicity can occur with anthracyclines, although this is the exception rather than the rule.

### 2. How Should this Patient’s Cardiac Dysfunction be Managed?

Because anthracycline-induced cardiotoxicity is dependent on the cumulative dose, discontinuation of treatment with anthracyclines must be considered. This decision must be based on a variety of factors, including the severity of left ventricular dysfunction and the availability of alternative, efficacious chemotherapeutic agents. As with trastuzumab-induced left ventricular dysfunction, patients with a decreased LVEF from anthracycline chemotherapy should receive standard heart failure treatment if there are no contraindications.

### 3. Could the Decrease in LVEF have been Prevented?

Recent studies have demonstrated that treatment with the ACE inhibitor, enalapril, prevents a decline in LVEF in high-risk patients [12, 13]. High risk was defined by an increase in the serum troponin I level following the initial treatment with doxorubicin, though it should be noted that clinical use of serum troponin I as a marker of risk in the context of anthracycline use has not yet been established [14]. In contrast, no benefit of treatment with enalapril was seen in low-risk patients. In addition to treatment with an ACE inhibitor, there is data to suggest that treatment with the beta-blocker carvedilol may prevent anthracycline-induced cardiotoxicity [12, 15].

### 4. Should Common Risk Factors for Cardiac Disease (e.g. Hypertension, Hyperlipidemia, Smoking, Family History) be Considered Contraindications to Anthracyclines?

In contrast to trastuzumab, risk factors for anthracycline-induced cardiotoxicity include the traditional cardiac risk factor, hypertension. Other risk factors include advanced age and the presence of pre-existing heart disease [6]. Although these factors increase the risk of anthracycline-induced cardiotoxicity, they should not preclude the use of anthracyclines in an individual with preserved left ventricular function. However, addition of an ACE inhibitor or beta-blocker to the patient’s regimen could be considered. In general, those patients who have pre-existing cardiac risk factors, including hypertension, valvular heart disease or pre-existing coronary artery disease, should undergo a formal baseline assessment of cardiac function.

### 5. If this Patient Were to Require Additional Treatment with Doxorubicin, what is the Threshold to Re-initiate Therapy in the Setting of Depressed Cardiac Function?

In patients with reduced LVEF, the decision to give anthracycline-based chemotherapy must weigh the benefit with respect to treating the cancer with the risk of exacerbating the heart failure. If anthracycline chemotherapy is associated with a high chance of curing the cancer, then a patient with a mild reduction in LVEF (40-49%), may be treated with the agent with serial assessments of left ventricular function performed prior to each cycle of chemotherapy [16]. 

## CLINICAL CASE 3

GF is a 62-year-old female with refractory/relapsed diffuse large B-cell lymphoma (DLBCL). Her past medical history was otherwise remarkable for type 2 diabetes and hypertension. The patient was first diagnosed with DLBCL eighteen months prior when she presented with enlarged sub-mandibular lymph nodes. She initially received standard first-line chemotherapy (CHOP-R: cyclophosphamide, doxorubicin, vincristine, prednisone, rituximab) for a total of six cycles, along with radiation to the neck. She achieved a complete remission, but about a year later she developed hip and flank pain and was ultimately found to have recurrence of disease. Additional chemotherapy (RICE: rituximab, ifosfamide, carboplatin,etoposide) was initiated, however the lymphoma proved resistant to therapy. Salvage chemotherapy (R-DHAX: cytarabine, oxaliplatin, dexamethasone, rituximab) was initiated, from which the patient achieved significant response. 

She was then deemed a candidate for bone marrow transplant, and ultimately underwent high-dose BEAM chemotherapy (carmustine, etoposide, cytarabine, melphelan) followed by autologous stem cell transplantation. The transplant was complicated by volume overload. Echocardiography at that time revealed an LVEF of 50%, concentric left ventricular hypertrophy, mild aortic insufficiency, and severe mitral regurgitation, which had not been present previously.

Following transplant, the patient did well for approximately three months until she was found to have recurrent disease on routine restaging PET scan.Chemotherapy was again initiated, with the CEPP-R regimen (cyclophosphamide, etoposide, procarbazine, prednisone, rituximab).

Several days after receiving chemotherapy, she was admitted to the hospital with lethargy and acute change in mental status. Her exam was remarkable for hypotension, confusion and sinus tachycardia. Head CT was unrevealing. Lab work-up revealed neutropenia, thrombocytopenia and an elevated troponin of 0.66 with an N-terminal pro-BNP of 34,400 pg/ml. Her EKG showed sinus tachycardia but no ischemic changes. Given concern for possible aspiration pneumonia, she was started on broad-spectrum antibiotics. 

Cardiology was subsequently consulted regarding the concern for myocardial infarction, both in terms of acute management and in terms of how this event might impact consideration of further chemotherapy for her DLBCL. Repeat echocardiography demonstrated a moderately dilated left atrium with a LVEF of 40%. There was normal right ventricular size and systolic function, as well as moderate aortic regurgitation and moderate to severe mitral regurgitation. There was no significant pericardial effusion. She was subsequently started on a beta-blocker, furosemide and an ACE inhibitor. 

## CARDIOLOGY CONSULT QUESTIONS

### 1. How Common are Cardiac Complications from Stem Cell Transplant? Does this Vary Between Autologous and Allogeneic Transplants, or Among Agents Used for Marrow Preparation (e.g. High-Dose Cyclophosphamide for Autologous Stem Cell Transplant)?

Cardiac complications from stem cell transplantation for hematologic malignancies are rare; however, agents used for the marrow preparation can have cardiac toxicities. For example, cyclophosphamide can cause a hemorrhagic pericarditis and myocarditis, which, although usually limited, can be life threatening [17].

### 2. Can this Patient’s Ischemic Episode be Attributed to Her Chemotherapy (e.g.Etoposide-Related Vasospasm)?

Although vasospasm caused by etoposide must be considered as a cause of the elevated serum troponin level, there were no electrocardiographic changes suggestive of vasospasm. Given the presentation suggestive of pneumonia, it is more likely that the patient had demand ischemia.

### 3. Could the Decline in Cardiac Function be Attributed to Any of the Prior Chemotherapeutic Agents?

The patient had previously received CHOP-R (cyclophosphamide, doxorubicin, vincristine, prednisone, and rituximab) prior to the bone marrow transplantation, which raises the possibility that the decrease in left ventricular function is related to treatment with anthracyclines.

### 4. Now that this Patient Appears to have had a Major Cardiac Complication in the Setting of Salvage Chemotherapy, what are the Implications for Further Treatment of her Lymphoma?

The presence of myocardial damage in the setting of demand ischemia raises the possibility that the patient has significant coronary artery disease. In addition, she has mildly reduced left ventricular function, the etiology of which (ischemic vs. chemotherapy-associated) has not been determined. As a result, she is certainly at increased risk for further decreases in left ventricular function with cardiotoxic chemotherapeutic regimens. Further risk stratification is somewhat problematic given her thrombocytopenia, which increases the risk of cardiac catheterization. After discussions between the oncologist and cardiologist, the prognosis of the patient from an oncologic standpoint was felt to be poor and further chemotherapy was not considered. The patient’s cardiac issues were treated medically with beta-blocker and ACE inhibitor.

## CONCLUSION

Cardiovascular complications of chemotherapy are fortunately uncommon. However, the development of cardiotoxicity during the course of chemotherapy can profoundly impact cancer treatment options, which may in turn affect overall cancer prognosis. Given the complexity of balancing oncologic risks with cardiac risks, many patients undergoing chemotherapeutic treatment for cancer may benefit from an integrated model of care between oncology and cardiology. The clinical vignettes presented here represent common scenarios in which multidisciplinary care is essential, including management of chemotherapy-related heart failure, prevention of cardiotoxicity, cardiac monitoring during chemotherapy and assessment of cardiac risk and prognosis in relation to selection of specific chemotherapy regimens. In short, the integration of oncology and cardiology can enhance overall care for many patients undergoing treatment for cancer, and should be considered as a standard approach for chemotherapy-related cardiotoxicity.
